# Fibroblast growth factor signalling influences homologous recombination-mediated DNA damage repair to promote drug resistance in ovarian cancer

**DOI:** 10.1038/s41416-022-01899-z

**Published:** 2022-07-01

**Authors:** Hugh A. Nicholson, Lynne Sawers, Rosemary G. Clarke, Kevin J. Hiom, Michelle J. Ferguson, Gillian Smith

**Affiliations:** 1 Division of Cellular Medicine, School of Medicine, University of Dundee, Ninewells Hospital and Medical School, Dundee, DD1 9SY UK; 2 Centre for Advanced Scientific Technologies, School of Life Sciences, University of Dundee, Dow Street, Dundee, DD1 5EH UK; 3 Tayside Cancer Centre, NHS Tayside, Ninewells Hospital and Medical School, Dundee, DD1 9SY UK

**Keywords:** Ovarian cancer, Growth factor signalling

## Abstract

**Background:**

Ovarian cancer patients frequently develop chemotherapy resistance, limiting treatment options. We have previously shown that individuality in fibroblast growth factor 1 (*FGF1*) expression influences survival and chemotherapy response.

**Methods:**

We used MTT assays to assess chemosensitivity to cisplatin and carboplatin following shRNA-mediated knockdown or heterologous over-expression of FGF1 (quantified by qRT-PCR and immunoblot analysis), and in combination with the FGFR inhibitors AZD4547 and SU5402, the ATM inhibitor KU55933 and DNA-PK inhibitor NU7026. Immunofluorescence microscopy was used to quantify the FGF1-dependent timecourse of replication protein A (RPA) and γH2AX foci formation.

**Results:**

Pharmacological inhibition of FGF signalling reversed drug resistance in immortalised cell lines and in primary cell lines from drug-resistant ovarian cancer patients, while FGF1 over-expression induced resistance. Ataxia telangiectasia mutated (ATM) phosphorylation, but not DNA adduct formation was FGF1 dependent, following cisplatin or carboplatin challenge. Combining platinum drugs with the ATM inhibitor KU55933, but not with the DNA-PK inhibitor NU7026 re-sensitised resistant cells. FGF1 expression influenced the timecourse of damage-induced RPA and γH2AX nuclear foci formation.

**Conclusion:**

Drug resistance arises from FGF1-mediated differential activation of high-fidelity homologous recombination DNA damage repair. FGFR and ATM inhibitors reverse platinum drug resistance, highlighting novel combination chemotherapy approaches for future clinical trial evaluation.

## Background

Ovarian cancer, the most deadly gynaecological malignancy, frequently presents when already advanced and adjuvant or neo-adjuvant chemotherapy combining cisplatin or, more frequently, carboplatin with paclitaxel is the most appropriate treatment [1]. Although many patients initially respond well, the development of treatment-limiting drug resistance is unfortunately inevitable in the majority of patients [2, 3]. There is therefore an urgent need to understand the molecular mechanisms underpinning drug resistance, and to use this information to develop clinical response biomarkers and propose novel combination chemotherapy regimens.

We have previously shown that individuality in fibroblast growth factor 1 (*FGF1*) expression significantly inversely influences both progression-free and overall survival in ovarian cancer patients [4]. *FGF1* expression is significantly increased in cisplatin-resistant cell line models, where *FGF1* knockdown in resistant A2780DPP cells re-sensitised to both cisplatin and carboplatin [4]. Consistent with our findings, others have confirmed that *FGF1* is more highly expressed in platinum-resistant compared to drug-sensitive ovarian cancers [5]. FGF1 is one of 22 fibroblast growth factors, related by sequence conservation and structural similarity, the majority of which drive autocrine signalling by binding to FGF receptors (FGFR1-4) to induce receptor tyrosine kinase signalling cascades. These intracellular cascades are activated by phosphorylation of the adaptor protein FGFR Substrate 2α (FRS2α) following CRK-like proto-oncogene recruitment to FGFR, which drives signalling through the PLCγ, MEKK, ERK1/2 and AKT pathways [6–13]. Dysregulated fibroblast growth factor signalling is frequently observed in many cancers [14]; however, the molecular mechanisms rationalising a role for the FGF signalling pathway in promoting drug resistance remain poorly characterised.

Cisplatin and carboplatin are actively transported into the cell by the copper transporter CTR1 [15–17]. Intracellular translocation results in spontaneous drug aquation [18]—both drugs then promote DNA damage induced by the formation of DNA interstrand cross-links that exceed cellular repair capacity [19]. This in turn forces cells to introduce DNA double-strand breaks (DSBs) [20], producing genomic instability. As maintenance of genome stability is fundamental to eukaryotic cell survival [21], cells have evolved highly conserved networks of DNA damage repair mechanisms [22]. DSBs are most commonly repaired by low-fidelity non-homologous end joining (NHEJ) or high-fidelity homologous recombination (HR) [23, 24]. NHEJ promotes direct ligation of broken DNA ends, whereas HR involves the replacement of damaged DNA using template sister chromatid sequences and is an essential defence against genome instability [25, 26]. Alterations to DNA damage repair mechanisms commonly induce resistance to DNA damaging drugs, including cisplatin and carboplatin [27]. For example, delayed engagement of HR results in toxic chromatid fusions [28], while compromised HR, for example as a result of inherited *BRCA1* or *BRCA2* mutations, confers sensitivity to poly-ADP ribose polymerase inhibitors including Olaparib [29, 30], and increases sensitivity to cisplatin and carboplatin chemotherapy in ovarian cancer patients [31].

An early step in HR is the focal recruitment of replication protein A (RPA), which protects 3’ single-stranded DNA overhangs from degradation [32] and promotes recruitment of RAD51 recombinase that induces sister chromatid invasion to facilitate homology-directed error-free repair [33]. HR-specific proteins are activated at DSB sites following the phosphorylation of the central regulator of DSB repair, ataxia telangiectasia mutated (ATM) [34–36]. ATM is an important regulator of DSB repair that phosphorylates multiple substrates [34], including itself at Ser1981 to induce its activation [35], and H2AX at Ser139 [36] to signal the presence of DSBs and instigate focal recruitment of crucial DNA damage repair proteins involved in homology-directed DSB repair [37].

Confirmation of a mechanistic link between FGF signalling and the DNA damage response would not only rationalise the influence of individuality in FGF1 expression on disease progression and chemotherapy response in ovarian cancer patients, but may additionally identify candidate resistance biomarkers and novel combination chemotherapy/resistance pathway inhibitor approaches for future clinical trial evaluation.

## Methods

### Cell culture

The A2780 cell line (chemosensitive, derived from an untreated ovarian cancer patient [38]), cisplatin-resistant derivative A2780DPP (A2780Cis [39]), and the SK-OV-3 cell line (platinum drug-sensitive ovarian adenocarcinoma cell line [40]) were purchased from the European Collection of Authenticated Cell Cultures. The CaOV3 cell line (platinum drug-sensitive epithelial ovarian cancer cell line derived from tumour tissue of a patient with adenocarcinoma of the ovary [41]) was a gift from Dr Jozien Helleman, Erasmus University, Netherlands. Cells were maintained in RPMI1640 medium (Gibco, Renfrewshire, UK) supplemented with 10% v/v foetal bovine serum (FBS) (Gibco, Renfrewshire, UK) and 1% v/v penicillin/streptomycin (Sigma Aldrich, Dorset, UK), with the addition of 1 µM cisplatin (Sigma Aldrich, Dorset, UK) to A2780DPP cells on every third passage. Further information summarising the genetic background of our ovarian cancer cell line panel is summarised in Supplementary Fig. 1. Human embryonic kidney 293T cells were a gift from Dr Laureano de la Vega, University of Dundee, and were maintained in DMEM medium (Gibco, Renfrewshire, UK) supplemented with 10% v/v FBS. All cell lines were maintained in 37 °C incubators supplemented with 5% CO_2_. Cell lines were authenticated by short tandem repeat profiling (Centre for Life, Newcastle, UK) and routinely tested and found to be negative for mycoplasma contamination (Lonza Biologics, Slough, UK).

### Cell culture of patient-derived cells

Patient ascites samples were collected following paracentesis and mixed 1:1 with ascites media (1:1 MCDB (Sigma Aldrich, Dorset, UK)/Media 199 (Gibco, Renfrewshire, UK), 10% v/v FBS, 1% v/v penicillin/streptomycin (Sigma Aldrich, Dorset, UK)) [42]. Cells were incubated for 3–5 days before differential trypsinisation (cells incubated in 1 mL trypsin for 30 s) to limit fibroblast contamination, then incubated for an additional 3 days before use in subsequent experiments.

### Lentiviral shRNA-mediated *FGF1* knockdown

shRNA constructs were prepared from bacterial glycerol stocks using midi-prep kits (Qiagen, Manchester, UK) according to the manufacturers’ instructions. 3 × 10^6^ HEK293T cells were transfected in 10 cm dishes using 5 µL Lipofectamine 2000 (Invitrogen, Renfrewshire, UK) with 5 µg of shRNA construct, one of several constructs previously evaluated to target *FGF1* (Sigma Aldrich, Dorset, UK; TRCN0000072524) [4], together with 3.2 µg psPAX2 packaging and 1.8 µg pMD2.G enveloping vectors (a gift from Dr Rita Moreno, University of Dundee) [43] in 3 mL Opti-MEM serum-free medium (Gibco) with 7 mL DMEM for 24 h before virus-containing supernatant was passed through a 0.45 µm filter (VWR, Leicestershire, UK). 2.5 × 10^5^ A2780DPP target cells seeded 24 h previously in six-well plates were then cultured for 24 h in virus-containing medium supplemented with 200 µg/mL hexadimethrine bromide (Sigma Aldrich, Dorset, UK), and transduced cells selected by 24 h incubation in RPMI supplemented with 2 µg/mL puromycin to create the A2780DPP FGF^KD^ cell line (Gibco, Renfrewshire, UK). A control cell line (A2780DPP EV) was similarly created, following transfection with the construct pLKO.1.

### siRNA-mediated *ATM* knockdown

2.5 × 10^5^ A2780DPP cells were seeded in six-well plates and incubated for 24 h before transient transfection using 5 µL Lipofectamine RNAiMAX (Thermo Fisher, Renfrewshire, UK) according to the manufacturers’ instructions with a final concentration of 5, 10 or 20 nM SMARTpool ON-TARGETplus *ATM*-targeted siRNA (L-003201-00-0005) or negative control non-targeted siRNA (D-001810-10-05, Horizon Discovery, Cambridge, UK) in Opti-MEM serum-free medium (Gibco, Renfrewshire, UK).

### FGF1 over-expression by plasmid transfection

2.5 × 10^5^ A2780 cells were seeded in six-well plates and pre-incubated for 24 h before transfection with either 1 µg pCMV3.1-EGFP-FGF1 plasmid (Sino Biological, Beijing, China) or 1 µg pCMV3.1-EGFP control plasmid (Sino Biological, Beijing, China) using 5 µL Lipofectamine 2000 (Invitrogen, Renfrewshire, UK) in 2 mL Opti-MEM reduced serum medium (Gibco, Renfrewshire, UK). Cells were incubated for 24 h in transfection mix and then selected with 5 µg/mL hygromycin (Gibco, Renfrewshire, UK) for 120 h.

### RNA extraction

Total RNA was extracted from 2.5 × 10^5^ cells using RNeasy Mini Kits (Qiagen, Manchester, UK), following the manufacturers’ guidelines, including an on-column DNase digestion (RNAs free DNase Kit, Qiagen, Manchester, UK). RNA yield and integrity were confirmed using a Nanodrop ND1000 spectrophotometer (Thermo Fisher, Renfrewshire, UK).

### qRT-PCR analysis

RNA was reverse transcribed into cDNA (4 ng/µL final concentration of RNA) using TaqMan Reverse Transcription Reagents Kit (Thermo Fisher, Renfrewshire, UK) according to the manufacturer’s instructions, replacing oligo dT with random hexamers. *FGF1* (Hs01092738_m1)*, ATM* (Hs00175892_m1) and *18S ribosomal RNA* expression (438839) was assessed in individual 20 µL reactions, combining 10 µL TaqMan universal master mix (Thermo Fisher, Renfrewshire, UK), 1 µL gene-specific probe, 1 µL cDNA and 8 µL nuclease-free water. Each reaction was performed in triplicate and run on the standard PCR programme (50 °C 2 min, 95 °C 10 min, and 40 cycles of 95 °C 15 s, 60 °C 1 min) on a QuantStudio5 qRT-PCR instrument (Thermo Fisher, Renfrewshire, UK). Baseline and threshold values were calculated automatically, and gene expression was quantified by cycle threshold (Ct) values, with relative gene expression comparing the Ct change in target gene and 18S rRNA control (ΔCt), as previously described [44]. Compound errors (s) were calculated using the formula *s* = ((standard deviation target gene)^2^ + (standard deviation 18S rRNA)^2^)^½^ (https://assets.thermofisher.com/TFS-Assets/LSG/manuals/cms_042380.pdf).

### Immunoblot analysis

2.5 × 10^5^ cells were seeded in six-well plates and incubated for 24 h prior to harvest or treatment with either an acute cisplatin challenge (3 µM) (Sigma Aldrich, prepared as a 2 mg/mL stock in sterile water), the FGFR inhibitors AZD4547 (ApexBio, Cambridge, UK, prepared as a 10 mM stock in DMSO) or SU5402 (Sigma Aldrich, Dorset, UK, prepared as a 10 mM stock in DMSO), or transfection as described above. Plates were placed on ice and cells were washed with ice-cold phosphate-buffered saline. Protein extracts were prepared in RIPA buffer (50 mM Tris-HCl (pH8), 150 mM NaCl, 0.1% SDS, 0.1% sodium deoxycholate, 1% NP-40, 2 mM EDTA, 2.1 g/L NaF), supplemented with EDTA-free protease inhibitor cocktail tablet (Sigma Aldrich, Dorset, UK) using a cell scraper. Lysates were collected following centrifugation at 2000 × g for 10 min at 4 °C, and protein concentrations were determined using the DC protein assay (BioRad, Hertfordshire, UK). Samples were diluted to 1 µg/µL with RIPA buffer, mixed with 10× sample reducing agent (500 mM dithiothreitol, Thermo Fisher, Renfrewshire, UK) and 4× BOLT sample buffer (Thermo Fisher, Renfrewshire, UK) and denatured at 100 °C for 10 min. Proteins were separated by electrophoresis in 12% lithium-dodecyl sulphate gels in Tris-glycine buffer (25 mM Tris pH8.3, 192 mM glycine, 0.1% SDS) with the addition of 500 µL NuPage antioxidant (Thermo Fisher, Renfrewshire, UK), then transferred to 0.45-µm-pore nitrocellulose membranes (GE Healthcare, Bucks, UK) in Tris-glycine-methanol buffer (25 mM Tris, 192 mM glycine, 20% methanol). Non-specific antibody binding was blocked by incubation in 5% non-fat dried milk in TBS-T (50 mM Tris (pH 7.9), 150 mM NaCl, 1% Tween-20), and membranes incubated overnight at 4 °C with either goat polyclonal anti-FGF1 (AF232, R&D Systems, Oxfordshire, UK, diluted 1:1000), rabbit polyclonal anti-GFP (G1544, Sigma Aldrich, diluted 1:2000) or anti-FRS2a (pY196) (PA5-64616 Thermo Fisher, diluted 1:1000), mouse monoclonal anti-FRS2α (MAB4-69, R&D Systems, diluted 1:1000), anti-β−Actin (sc-47778, Santa Cruz Biotechnology, Heidelberg, Germany, diluted 1:1000), or anti-γH2AX (05-636, Merck Life Science, Darmstadt, Germany), or rabbit monoclonal anti-ATM (S1981) (5883T, Cell Signaling Technology, Danvers, MA, USA, diluted 1:500), or anti-ATM (2873T, Cell Signaling Technology, diluted 1:1000). Membranes were washed in TBS-T and incubated for 1 h in HRP-conjugated rabbit anti-goat polyclonal secondary antibody (FGF1; 170-1034, BioRad, diluted 1:2000), HRP-conjugated goat anti-mouse polyclonal secondary antibody (FRS2α, β−Actin, γH2AX; 170-6516, BioRad, diluted 1:2000), or HRP-conjugated goat anti-rabbit polyclonal secondary antibody (GFP, FRS2α (Y196), ATM (S1981), ATM; 170-6515, BioRad, diluted 1:2000). Immunoblots were developed using Amersham enhanced chemiluminescence kit (GE Healthcare, Buckinghamshire, UK) using Amersham high-performance Hyperfilms (GE Healthcare, Buckinghamshire, UK).

### Slot blot analysis

2 × 10^5^ cells/well were seeded in six-well plates and incubated overnight. Cells were synchronised by treatment with 2 mM thymidine for 24 h before treatment with 3 µM cisplatin or 10 µM carboplatin for 3 h, harvested by trypsinisation and genomic DNA extracted using GeneJet Genomic DNA purification kit (Thermo Fisher, Renfrewshire, UK) according to the manufacturers’ instructions. DNA was diluted to 100 ng/150 µL in 0.4 M NaOH/10 mM EDTA and boiled at 100 °C for 10 min before immediate incubation on ice and the addition of ice-cold 150 µL 2 M NH_4_CH_3_CO_2_. DNA samples were bound to Protran nitrocellulose membranes (BioRad, Hertfordshire, UK) (pre-incubated in 0.9 M NaCl, 90 mM sodium citrate, pH 7.0) by vacuum manifold filtration and membranes washed with 0.3 M NaCl, 30 mM sodium citrate, air-dried for 4 h at room temperature then rehydrated in PBS-0.1% Tween, with non-specific antigen binding blocked with 5% BSA, 0.05% w/v NaN_3_. Membranes were incubated in anti-platinum-modified DNA Ab (1:1000; Abcam, Cambridge, UK) in 5% BSA, 0.05% w/v NaN_3_ at 4 °C overnight, washed in PBS-0.1% Tween, and incubated in anti-Rat IgG HRP, 1:2000, 5% BSA, 0.05% w/v NaN_3_, (BioRad, Herts, UK) for 1 h. Membranes were washed in PBS-0.1% Tween and slot blots were developed using Amersham enhanced chemiluminescence kit (GE Healthcare, Buckinghamshire, UK) using Amersham high-performance Hyperfilms (GE Healthcare, Buckinghamshire, UK).

### In vitro chemosensitivity assays

3-(4,5-Dimethylthiazol-2-yl)-2,5-diphenyltetrazolium bromide (MTT) assays [45] were used to compare the chemosensitivity of cell lines. Cells were seeded in 96-well plates (5000 cells per well in 100 µL media). Cells were untreated or treated in triplicate with vehicle or with serial dilutions of cisplatin or carboplatin at concentrations relevant to typical ovarian cancer patient peak plasma levels (range 0–200%; cisplatin (0–25.33 µM), carboplatin (0–85.12 µM) [46], and in combination with FGFR, ATM or DNA-PK inhibitors (10 µM SU5402, AZD4547; 10 µM KU55933, 2 µM NU7026). Following 72 h (immortalised cell lines, as previously described [4, 45]) or 144 h (to allow for slower growth of primary patient-derived cells), media and drugs were removed and 100 µL 0.5 mg/mL MTT (Alfa Aesar, Lancashire, UK) solution (MTT in phenol-free RPMI, Gibco, Renfrewshire, UK) added and incubated for 3 h at 37 °C, 5% CO_2_. MTT solution was removed, and formazan crystals formed by viable cells were solubilised in 100 µL DMSO and quantified by absorbance at 570 nm. The percentage of viable cells remaining following drug treatment was calculated as a percentage of vehicle-treated control cells and associated IC_50_ values were calculated from log dose-response curves using Prism 9 software (GraphPad Software, Inc., La Jolla, CA, USA).

### Immunofluorescence microscopy

Immunofluorescence microscopy was used to assess Pt-DNA adduct formation in ovarian cancer cells with different levels of *FGF1* expression and HR-specific nuclear foci formation in response to cisplatin-induced DNA damage. 2 × 10^6^ cells were seeded per well of a six-well plate containing a #1.5 glass coverslip ø22mm (VWR, Leicestershire, UK) and incubated overnight. Cells were synchronised with 2 mM thymidine (Alfa Aesar, Lancashire, UK) for 24 h and, following thymidine washout, treated for 3 h with 3 µM cisplatin, then fixed immediately to assess adduct formation or washed in pre-warmed PBS and incubated over a 24 h (RPA, RAD51) or 36 h (γH2AX) timecourse with cells fixed at appropriate timepoints. To fix cells, coverslips were washed in PBS, incubated for 10 min in 4% paraformaldehyde/NaOH (pH 7.2), then washed in PBS and permeabilised in KCM buffer (120 mM KCl, 20 mM NaCl, 10 mM Tris-HCl, pH 7.5, 0.1% v/v Triton-X100) for 10 min. Non-specific antigen binding was blocked by incubation in blocking buffer (20 mM Tris-HCl pH 7.5, 150 mM NaCl, 2% w/v BSA, 2% w/v fish gelatine, 0.1% Triton-X100) for 20 min at 37 °C, prior to incubation in primary antibody (cisplatin-modified DNA, rat monoclonal CP9/19, ab103261, Abcam (Cambridge, UK), diluted 1:1000; RPA, mouse monoclonal, RPA34-19, NA18 Calbiochem (Dorset, UK), diluted 1:1000; RAD51, rabbit monoclonal, EPR4030 (3) ab133534, Abcam, Cambridge, UK, diluted 1:1000; γH2AX, mouse monoclonal JBW301, 05-636, Merck (Dorset, UK), diluted 1:1000) in blocking buffer at 4 °C overnight in a damp chamber. Coverslips were washed in PBS-T (PBS, 0.1% Tween-20) and then incubated in secondary AlexaFluor-conjugated antibody (cisplatin-modified DNA, donkey anti-rat AlexaFluor488 A21208, Thermo Fisher; RPA and γH2AX goat anti-mouse AlexaFluor488 A11001, Thermo Fisher; RAD51 recombinase, donkey anti-rabbit AlexaFluor594 R37119, Thermo Fisher; all 1:2000) and DAPI (1:3000) in blocking buffer for 2 h in a damp chamber at RT. Coverslips were washed in PBS-T, air-dried for 3 h and mounted to SuperFrostPlus microscope slides (VWR, Leicestershire, UK) with the addition of 5 µL Prolong Gold anti-fade reagent (Thermo Fisher, Renfrewshire, UK). Confocal microscopy was performed on each coverslip using an LSM-710 microscope (Carl Zeiss, Oberkochen, Germany), using an inverted lens at ×40 magnification using oil immersion. Images were taken in Z-stacks where the maximum intensity projection was used for subsequent image analysis in OMERO insight (University of Dundee, UK), with a minimum of 40 cells counted in each experiment. Nuclear foci were quantified using Fiji Image J v2.0 open-source image processing package using Duke University Protocol (Durham, NC, USA) and data processed using Prism 9 software.

### Statistical analysis

Statistical comparisons of data were performed using two-tailed Student’s *t*-tests for pairwise comparisons or one-way analysis of variance for multiple comparisons using GraphPad Prism 9, with *p* values <0.05 considered to represent statistical significance (*p < 0.05, **p < 0.01, ***p < 0.001).

## Results

### FGF1 influences sensitivity to cisplatin and carboplatin in ovarian cancer cells

**Fig. 1:**
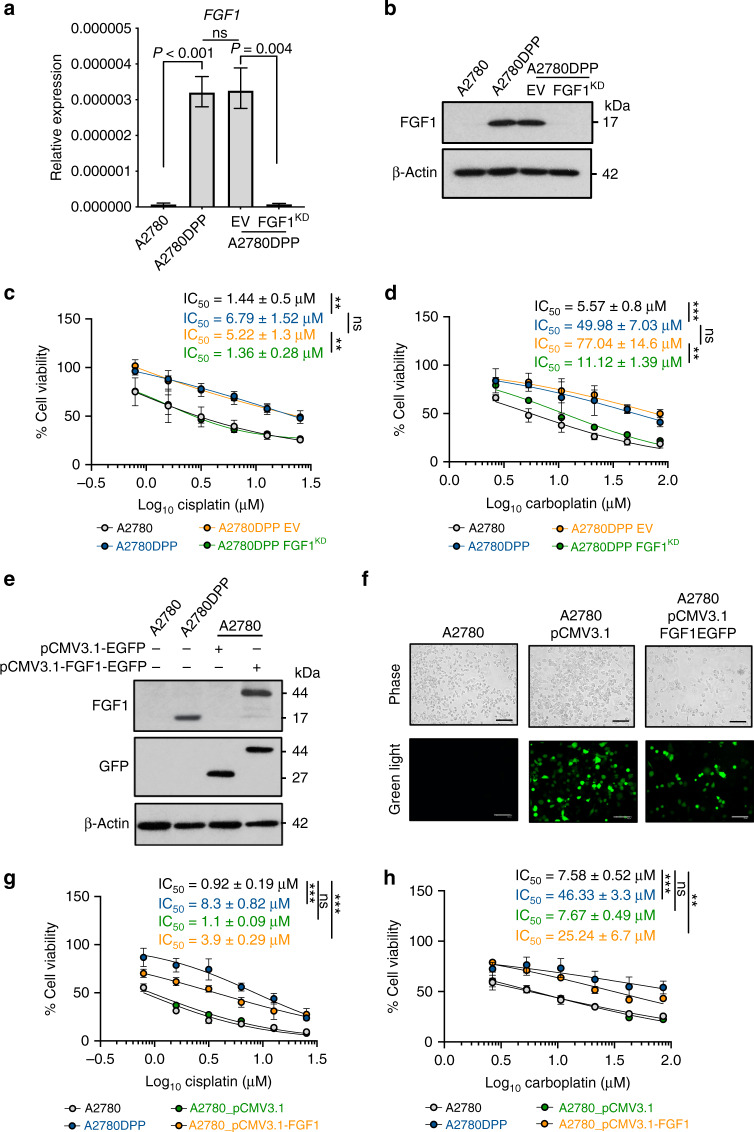
FGF1 influences sensitivity to cisplatin and carboplatin in ovarian cancer cells. A2780DPP cells were lentivirally transduced with an shRNA construct targeting *FGF1* or an empty vector control and *FGF1* expression assessed using **a** qRT-PCR analysis and **b** immunoblot analysis (EV empty vector). MTT assays were used to assess the effect of *FGF1* knockdown on sensitivity to **c** cisplatin (0–25.33 µM) and **d** carboplatin (0–85.12 µM). *FGF1* was overexpressed in A2780 cells using an FGF1-EGFP fusion plasmid and **e** immunoblot analysis and **f** green fluorescence imaging used to confirm FGF1 expression. MTT assays were used to assess the influence of *FGF1* over-expression on sensitivity to **g** cisplatin (0–25.33 µM) and **h** carboplatin (0–85.12 µM). Results represent three independent experiments. Pairwise comparisons of mean IC_50_ values and relative gene expression were performed using Student’s *t*-tests. Scale bar = 100 µm. *p < 0.05, **p < 0.01, ***p < 0.001.

To extend our previous analysis [4], we used lentivirus to create an A2780DPP derivative cell line with shRNA-mediated stable *FGF1* knockdown (A2780DPP FGF1^KD^). *FGF1* knockdown (274.4-fold change in expression, *p* = 0.004) was confirmed by qRT-PCR (Fig. 1a) and immunoblot (Fig. 1b) analysis and was stable for more than 20 passages (data not shown). Loss of *FGF1* expression in A2780DPP cells again re-sensitised drug-resistant cells to cisplatin (3.84-fold change in IC_50,_ *p* = 0.007, Fig. 1c) and carboplatin (6.9-fold change in IC_50,_ *p* = 0.002, Fig. 1d), assessed by MTT assay. We further confirmed that *FGF1* knockdown influenced colony formation following cisplatin and carboplatin challenge by clonogenicity assay (Supplementary Fig. 2).

To complement these experiments, *FGF1* expression was increased in drug-sensitive A2780 cells by transfection with a dual GFP/*FGF1* expression plasmid. Increased expression of both FGF1 and GFP was confirmed by immunoblot analysis (Fig. 1e) and green fluorescence microscopy (Fig. 1f). As predicted, increased *FGF1* expression induced cisplatin (3.54-fold change in IC_50,_ *p* < 0.001, Fig. 1g) and carboplatin (3.29-fold change in IC_50,_ *p* = 0.002, Fig. 1h) resistance in A2780 cells. Similarly, resistance to cisplatin and carboplatin was induced by heterologous *FGF1* expression in chemonaive SK-OV-3 and CaOV3 ovarian cancer cells (Supplementary Fig. 3).

### FGF receptor inhibition re-sensitises A2780DPP cells to cisplatin and carboplatin

**Fig. 2:**
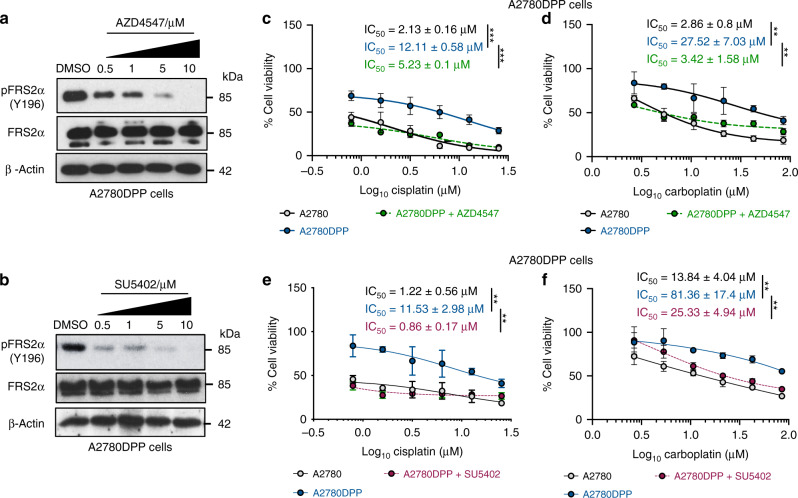
FGF receptor inhibition re-sensitises A2780DPP cells to cisplatin and carboplatin. Immunoblot analysis was used to assess blockade of the FGF signalling pathway in A2780DPP cells in response to **a** AZD4547 (0–10 µM) and **b** SU5402 (0–10 µM). MTT assays were used to investigate the viability of A2780DPP cells following co-treatment with 10 µM AZD4547 or 10 µM SU5402 and **c**, **d** cisplatin (0–25.33 µM) and **e**, **f** carboplatin (0–85.12 µM). Results represent three independent experiments. Pairwise comparisons of mean IC_50_ values were calculated by Student’s *t*-tests. *p < 0.05, **p < 0.01, ***p < 0.001. Ns not significant.

To investigate whether pharmacological inhibition of the FGF signalling pathway in A2780DPP cells recapitulates *FGF1* knockdown, the Type I inhibitors AZD4547 and SU5402 which inhibit all four FGF receptors were used. Treatment of A2780DPP cells with AZD4547 (Fig. 2a) and SU5402 (Fig. 2b) blocked FGFR signalling in a concentration-dependent manner, confirmed using immunoblot analysis to assess phosphorylation of FGF Receptor Substrate 2α (FRS2α, Y196) as a surrogate for pathway activity [13]. Co-treatment with AZD4547 re-sensitised A2780DPP cells to cisplatin (fold change IC_50_ = 2.32, *p* < 0.001, Fig. 2c) and carboplatin (fold change IC_50_ = 8.05, *p* = 0.004, Fig. 2d), as did co-treatment with SU5402 with cisplatin (fold change IC_50_ = 13.42, *p* = 0.004, Fig. 2e) and carboplatin (fold change IC_50_ = 3.19, *p* = 0.006, Fig. 2f), assessed by MTT assays. Neither inhibitor was independently toxic at the concentration used (data not shown).

### FGFR inhibitors reverse drug resistance in ascites-derived primary cell lines

**Fig. 3:**
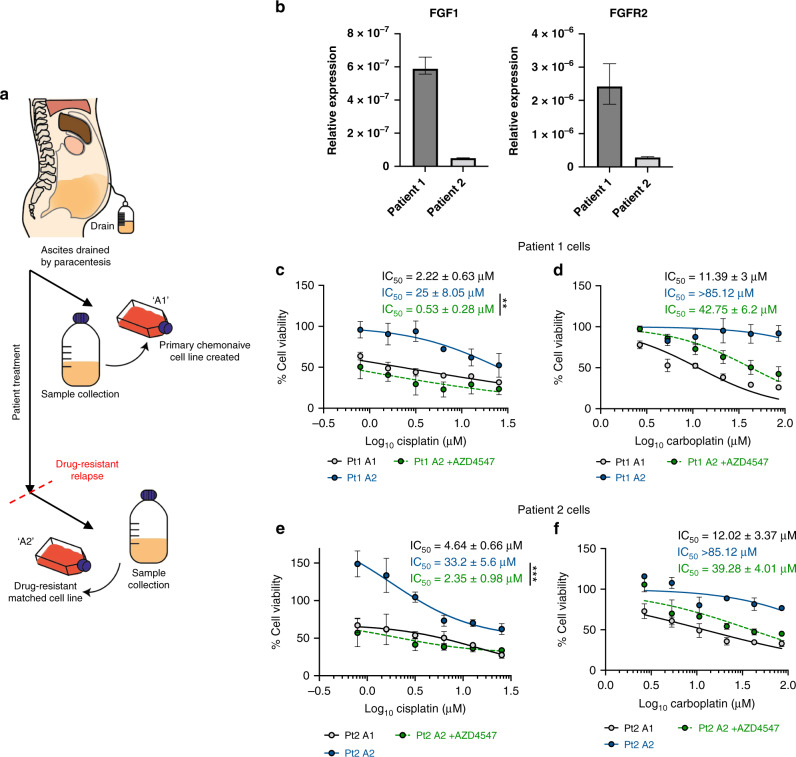
FGFR inhibitors reverse drug resistance in ascites-derived primary cell lines. **a** The Dundee Ovarian Cancer Study; ‘A1’ sample = chemonaïve, ‘A2’ sample = disease relapse. **b** *FGF1* and *FGFR2* gene expression in chemoresistant ovarian cancer patient-derived cells was assessed by qRT-PCR analysis. MTT assays were used to compare viability of chemonaive cells, and cells from drug-resistant relapse samples from two patients in the presence or absence of combination treatments with 10 µM AZD4547 and **c**, **e** cisplatin (0–25.33 µM) or **d**, **f** carboplatin (0–85.12 µM). Pairwise comparisons of mean IC_50_ values and relative gene expression were calculated by Student’s *t*-tests. *p < 0.05, **p < 0.01, ***p < 0.001. Ns not significant.

The Dundee Ovarian Cancer Study (DOCS) allows us to compare chemosensitivity and investigate associated resistance mechanisms in primary ascites-derived cell lines from chemotherapy naive drug-sensitive and matched drug-resistant ovarian cancer patients (Fig. 3a). To investigate whether FGFR inhibition also influenced cisplatin and carboplatin sensitivity in clinical samples in which the expression of both FGF1 and FGFR2 had been confirmed by qRT-PCR analysis (Fig. 3b), MTT assays were again used to assess chemosensitivity in two matched sample pairs before and after treatment with AZD4547, an ATP-competitive FGFR inhibitor, which was less toxic than SU5402 in previous clinical trials [47]. Both patients were initially clinically sensitive to cisplatin and carboplatin at disease presentation and relapsed with treatment-resistant disease. Although the primary ascites-derived cell line from Patient 1 showed higher expression of both FGF1 and FGFR2 than Patient 2, 10 µM AZD4547 in combination with either cisplatin (Fig. 3c, e) or carboplatin (Fig. 3d, f) again re-sensitised both drug-resistant cell lines—Patient 1 cisplatin fold change IC_50_ = 47.17, *p* = 0.006 and carboplatin fold change IC_50_ > 2, and Patient 2 cisplatin fold change IC_50_ = 14.13, *p* < 0.001 and carboplatin fold change IC_50_ > 2.17.

### Platinum-induced DNA adduct formation is independent of FGF1 expression

**Fig. 4:**
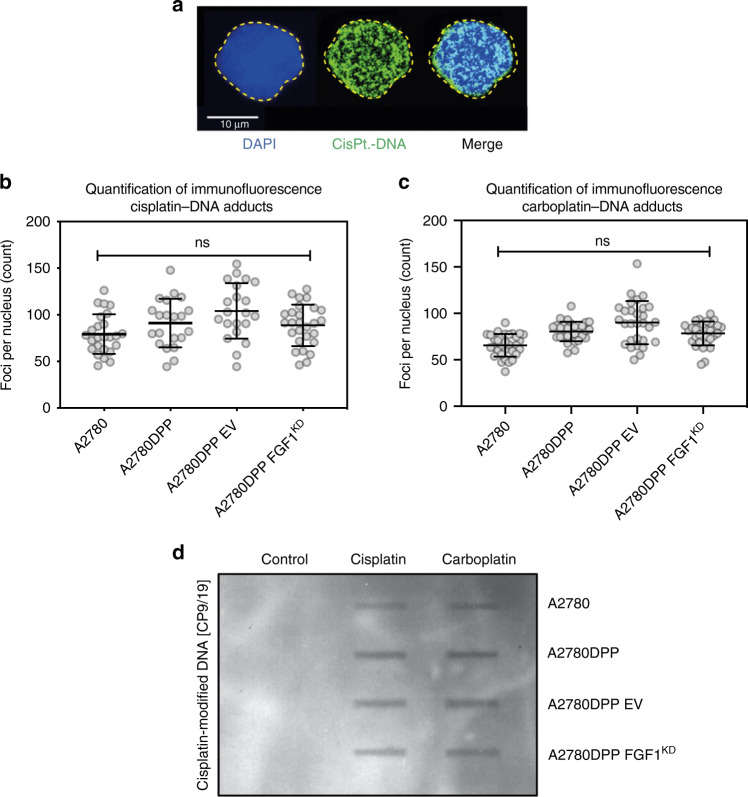
Platinum-induced DNA adduct formation is independent of FGF1 expression. Cells were thymidine synchronised (2 mM, 24 h) prior to cisplatin challenge (3 µM cisplatin for 3 h). Immunofluorescence microscopy was used to detect platinum-DNA adducts using a CP9/19 cisplatin-modified DNA antibody. A representative maximum intensity projection of a stained cell is illustrated in **a**, where DAPI was used as a nuclear stain, and the yellow line represents the nuclear boundary. Platinum-DNA adduct formation was evaluated in response to 3 µM cisplatin and 10 µM carboplatin by immunofluorescence (**b**, **c**) and slot blot (**d**) analysis. Results are illustrative of three independent experiments. Foci formation was compared by one-way ANOVA. Scale bar = 100 µm. *p < 0.05, **p < 0.01, ***p < 0.001. Ns not significant, EV empty vector.

Following CTR1-mediated influx, cisplatin and carboplatin kill cells principally by crosslinking to and forming adducts between opposing DNA strands [15–17, 19]. *CTR1* expression was not significantly different in A2780 and A2780DPP cells, and was not FGF1-dependent (data not shown). We then used immunofluorescence to quantify *cis*-[Pt(NH_3_)_2_]^2+^-DNA adduct formation in synchronised cells with different levels of *FGF1* expression following 3 μM cisplatin or carboplatin challenge, using an antibody which detects platinum-modified DNA structures (Fig. 4a). FGF1 expression did not significantly influence cisplatin or carboplatin-induced DNA adduct formation, assessed both by immunofluorescence (Fig. 4b, c) and by slot blot analysis (Fig. 4d).

### Double-strand break detection is influenced by FGF1 expression

**Fig. 5:**
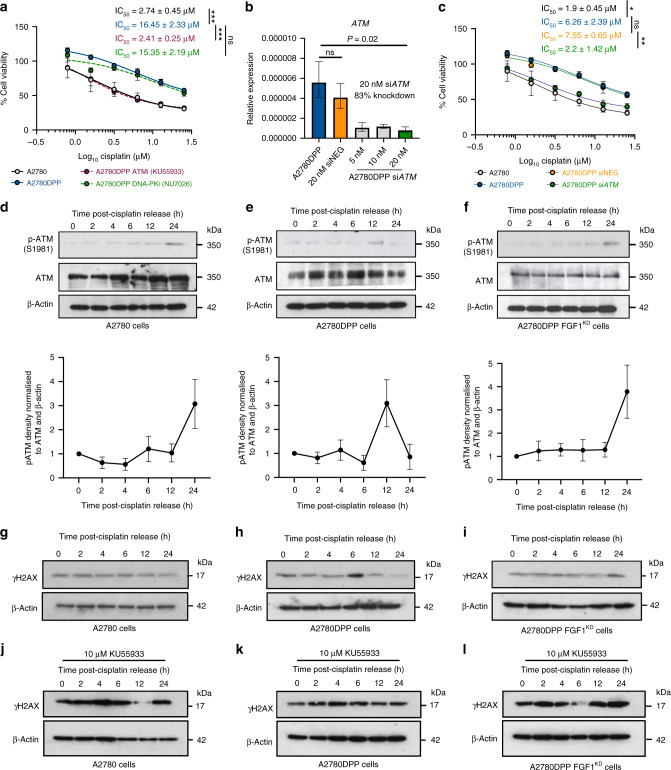
Double-strand break detection is influenced by FGF1 expression. **a** MTT assays were used to compare viability of A2780, A2780DPP and A2780DPP cells treated with 10 µM KU55933 (ATMi) or 2 µM NU7026 (DNA-PKi) in response to cisplatin (0–25.33 µM). **b** qRT-PCR analysis was used to assess *ATM* expression relative to 18S rRNA in response to 5 nM, 10 nM and 20 nM *ATM* siRNA (±compound SD). **c** MTT assays were used to compare the viability of A2780, A2780DPP and A2780DPP cells transfected with 20 nM siRNA scrambled negative control or 20 nM si*ATM* in response to cisplatin (0–25.33 µM). Representative immunoblot analysis comparing the phosphorylation of ATM (S1981) over indicated timepoints following 3 h treatment with 3 µM cisplatin in thymidine synchronised cells (2 mM, 24 h) in **d** A2780, **e** A2780DPP and **f** A2780DPP FGF1^KD^ cells. A timecourse of FGF1-dependency H2AX (S139; γH2AX) phosphorylation was investigated before (**g**–**i**) and following treatment with 10 µM KU55933 (ATMi) (**j**–**l**) in A2780, A2780DPP and A2780DPP FGF1^KD^ cells. Results are illustrative of four repeat experiments. Pairwise comparisons of mean IC_50_ values and relative gene expression were calculated by Student’s *t*-tests. Scale bar = 100 µm. *p < 0.05, **p < 0.01, ***p < 0.001. Ns not significant.

As drug influx and Pt-DNA adduct formation were not FGF1-dependent, we next investigated whether FGF1 could influence the DNA damage repair response. Inhibition of the NHEJ pathway with 2 µM NU7026 DNA-PK inhibitor (DNA-PKi) did not influence cisplatin sensitivity in A2780DPP cells (Fig. 5a). Conversely, inhibition of ATM with 10 µM KU55933 (ATMi) completely re-sensitised A2780DPP cells to cisplatin (Fig. 5a), suggesting that the HR pathway may be more important to the drug-resistant phenotype of these cells. Again, neither inhibitor was independently toxic at the concentrations used (data not shown). To confirm that chemosensitivity was ATM-dependent, siRNA-mediated *ATM* depletion was optimised in A2780DPP cells (Fig. 5b). Following siRNA knockdown, A2780DPP cells were re-sensitised to cisplatin (fold change IC_50_ = 3.43, *p* = 0.004; Fig. 5c), assessed by MTT assay. We next used immunoblot analysis to investigate whether ATM activation (Ser1981 phosphorylation) in response to DNA damage (3 μM cisplatin challenge) was FGF1-dependent. A2780 cells induced maximal ATM phosphorylation 24 h following cisplatin challenge (Fig. 5d). In contrast, maximal ATM phosphorylation occurred 12 h earlier in A2780DPP cells (Fig. 5e), a phenotype reversible by *FGF1* knockdown (Fig. 5f). Similar FGF1 dependency was observed for γH2AX expression. A2780 cells are sensitive to platinum drugs, and show basal γH2AX expression following cisplatin challenge (Fig. 5g). In contrast, resistant A2780DPP cells show a more transient γH2AX induction, 6 h following drug challenge (Fig. 5h), while re-sensitised A2780DPP FGF1^KD^ cells delay γH2AX induction to 24 h (Fig. 5i), consistent with the FGF1-dependent p-ATM expression described above. Dependency of the DNA damage response was further confirmed by combining cisplatin challenge with the ATMi inhibitor KU55933 (Fig. 5j–l). KU55933 inhibited γH2AX phosphorylation 12 h following treatment in A2780 cells but not in A2780DPP cells, while inhibition was restored and phosphorylation inhibited 6 h following treatment in A2780DPP FGF1^KD^ cells.

### FGF1 promotes HR pathway activation in response to cisplatin

**Fig. 6:**
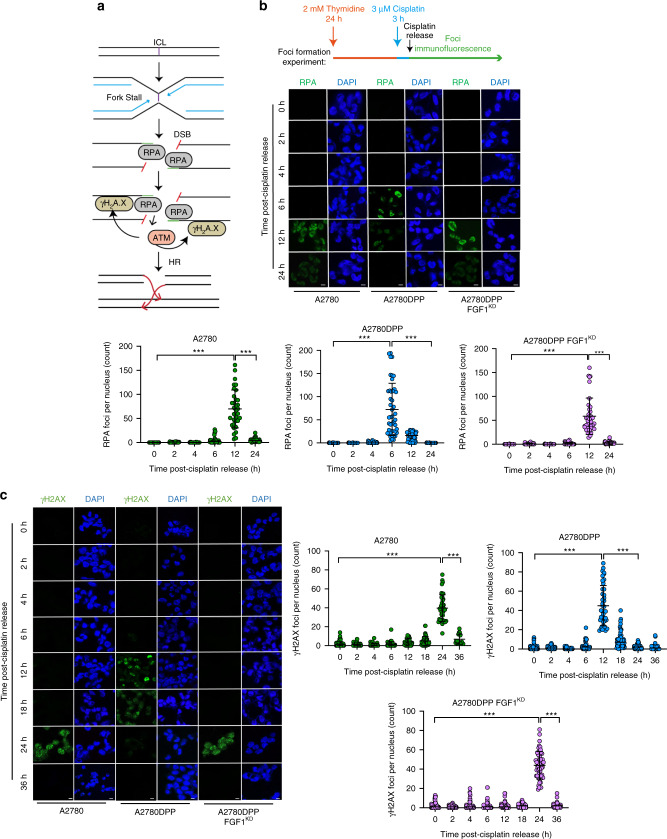
FGF1 promotes HR pathway activation in response to cisplatin. **a** Graphical abstract of HR-mediated DNA damage repair. ICL interstrand crosslink, DSB double-strand break, RPA replication protein A, ATM ataxia telangiectasia mutated, HR homologous recombination. Immunofluorescence and confocal microscopy were used to compare the formation and dissolution of **b** RPA foci and **c** γH2AX foci following 3 µM cisplatin challenge, following thymidine synchronisation (2 mM, 24 h). Typical representative maximum intensity projections are displayed. DAPI was used as a nuclear stain. *n* > 40 cells per treatment. Foci formation was compared by one-way ANOVA. ***p < 0.001. Scale bar = 20 µm.

HR is initiated by focal recruitment of RPA and γH2AX, which recruits DSB repair proteins [37], inducing sister chromatid invasion to facilitate homology-directed error-free DSB repair [33] (Fig. 6a). Three micromolar cisplatin challenge was again used to induce DSB formation in synchronised cells, and immunofluorescence was used to assess the formation of RPA and γH2AX foci in A2780, A2780DPP and A2780DPP FGF1^KD^ cells. In A2780 cells, RPA foci formed 12 h after cisplatin challenge. In contrast, foci were induced at 6 h in A2780DPP cells, with foci formation delayed to 12 h in A2780DPP FGF1^KD^ cells (Fig. 6b). Similarly, γH2AX foci formation was observed 24 h following cisplatin challenge in A2780 cells, at 12 h in A2780DPP cells, with foci formation delayed to 24 h in A2780DPP FGF1^KD^ cells (Fig. 6c), with a similar timecourse of FGF1-dependent RAD51 recombinase foci formation observed (Supplementary Fig. 4).

Finally, to allow us to consider whether our observed FGF1-mediated chemosensitivity and DNA damage response changes were influenced by altered cell cycle parameters in A2780, A2780DPP and A2780DPP FGF1^KD^ cells, we used flow cytometry to compare cell cycle profiles (a typical scatter plot, gating strategy and cell cycle distribution is illustrated in Supplementary Fig. 5a). While we saw a consistent modest increase in cells in S phase comparing the A2780 and A2780DPP cell lines (33.8% and 39.0%, respectively, *p* = 0.002), with a corresponding decrease in G1 cells (50.5% and 41.3%, respectively, *p* = 0.005), these changes were not FGF1-dependent, as FGF1 knockdown in A2780DPP cells did not have a significant influence on S and G1 phase cell numbers (Supplementary Fig. 5b–d, summarised in Panel e). In contrast, G2 cell numbers were FGF1 dependent, with a significant increase in A2780DPP cells compared to A2780 control cells (% increase 140.3, *p* = 0.003), which was significantly reduced following FGF1 knockdown (% reduction 82.3, *p* = 0.02, Supplementary Fig. 5f).

Taken together, our data therefore suggest that FGF1-induced cisplatin and carboplatin resistance is mediated by differential ATM phosphorylation and associated FGF1-dependent HR-mediated DSB repair.

## Discussion

Response to first-line chemotherapy, an important determinant of clinical outcome, is frequently compromised by the development of drug-resistant disease in ovarian cancer patients [2, 3, 48]. We have previously shown that *FGF1* expression modifies chemosensitivity to cisplatin and carboplatin in multiple ovarian cancer cell lines [4], consistent with data from Ryner et al. [5], describing increased expression of FGF pathway genes in the reactive stroma in chemoresistant ovarian cancer patients. We now confirm that introduction of FGF1 to drug-sensitive cells confers resistance, and that resistance is reversible by co-administration of pharmacological FGFR inhibitors in both immortalised cell lines and primary ascites-derived cell lines from drug-resistant ovarian cancer patients. Drug-resistant primary cell lines were re-sensitised by combination treatment with the FGFR inhibitor AZD4547—we prioritise extension of this clinical series as our DOCS study matures, and suggest that assessment of individuality in tumour and/or ascites FGF1 expression may have future clinical utility in patient selection for combination chemotherapy/FGFR inhibitor treatment. Importantly, AZD4547 is an orally bioavailable and well-tolerated Type I FGFR-specific inhibitor that has progressed to Phase II clinical trials for FGFR-positive squamous cell lung cancer (NCT02965378), breast, lung, and stomach cancer (NCT01795768) and solid tumours, lymphomas, and multiple myelomas (NCT02465060).

Platinum drug resistance has previously been reported to result from loss of drug-target interactions, due to either reduced intracellular drug accumulation (decreased drug influx or increased drug efflux) [49, 50], or from increased cytoplasmic drug sequestration [51]. In contrast, our data confirm that increased FGF1 expression confers drug resistance but does not directly influence Pt-DNA adduct formation, instead influencing the timecourse of HR-mediated DNA damage repair. Consistent with this hypothesis, previous studies have confirmed that platinum-DNA adducts are removed more rapidly in cisplatin-resistant ovarian cancer cells than in paired sensitive cell lines [52, 53]. We suggest that FGF1 influences DNA damage sensing and induces drug resistance by focal recruitment of the ssDNA protective protein RPA and associated phosphorylation of ATM, a kinase with a critical role in DSB sensing [36]. Differential FGF1-dependent ATM phosphorylation influences the kinetics of high-fidelity HR DNA damage repair activation, in preference to low-fidelity DSB repair by NHEJ, initiated by the binding of Ku70/80 heterodimers to blunt DNA ends [54]. ATM additionally promotes the removal of Ku heterodimers to inhibit NHEJ [55] and prevents the formation of NHEJ-induced chromatid fusions [28]. Our data therefore suggest that ATM is an important intermediate in the FGF1 signalling pathway, directly linking FGF signalling to HR-mediated DNA damage repair. As delayed engagement of HR results in the formation of toxic intermediates that promote apoptosis [28], we further describe an important role for FGF1 in accelerating HR pathway activation, which rationalises the resultant drug-resistant phenotype. It remains to be confirmed, however, whether DSBs formed by cisplatin-DNA adducts are induced, detected or repaired in an FGF1-dependent manner, and whether differential FGF1 expression influences the kinetics of adduct formation. FGF1-dependent RPA foci formation, however, suggests that FGF1 either promotes the formation or detection of DSBs. Consistent with our data, recent evidence in the gastrointestinal stromal tumour cell line T-1R shows that the alternative FGFR1-3 inhibitor BGJ398 (Infigratinib) or knockdown of *FGFR2* attenuates DSB repair by HR and not by NHEJ [56]. We have not investigated the influence of FGF1 on other components of the HR pathway, for example, MRE11 and RAD51, in the current study - but highlight this as an interesting area for future focus, as differential expression of both genes has also previously been associated with platinum drug chemosensitivity in various cancers [57, 58], with RAD51 particularly related to platinum drug resistance in ovarian cancer following the restoration of genomic integrity through secondary reversion mutations [59]. It was particularly interesting to note that, although the effects are modest, we observed an FGF1-dependent increase in the G2 cell population in resistant A2780DPP cells. Although FGF1-dependent cell cycle regulation has not previously been associated with drug resistance, FGF1 has previously been shown to induce an ATM-dependent G2 arrest in RCS chondrocytes [60], and G2 arrest has previously been associated with cisplatin resistance in ovarian cell lines [61] and in other in cancer cell lines including lung adenocarcinoma [62] and gastric cancer [63]. We are therefore currently optimising experimental protocols to allow us to investigate the influence of cell cycle kinetics on the evolution of drug resistance in our DOCS study primary ovarian cancer cell models.

We have further shown that ATM inhibitors can re-sensitise immortalised platinum-resistant ovarian cancer cells, suggesting that ATM inhibition may be an additional novel candidate combination chemotherapy approach. We highlight the need to extend our analysis to clinical samples, and note that the orally bioavailable ATM inhibitor AZD0156 is currently in clinical trials in combination with other cytotoxic chemotherapies, including olaparib and irinotecan (NCT02588105). To further validate our hypothesis that the FGF1-mediated HR DNA damage response pathway is ATM-dependent, we additionally highlight the importance of future experiments to confirm that γH2AX foci formation is also reduced in the presence of FGFR inhibitors.

In summary, we therefore extend our previous data describing the promotion of drug resistance by a platinum chemotherapy-induced increase in FGF1 expression, and highlight that pharmacological inhibition of FGF signalling can re-sensitise both immortalised drug-resistant cells and primary cell lines from drug-resistant ovarian cancer patients. We describe a novel ATM kinase-mediated mechanism linking FGF and DNA damage response signalling, with cisplatin and carboplatin chemosensitivity influenced by FGF1-dependent regulation of the HR DNA damage response pathway, where FGF1 accelerates the kinetics of HR-mediated DNA damage repair.

## Supplementary information

Supplementary Information

## Data Availability

All data generated or analysed during this study are included in this published article [and associated Supplementary information files].
