# Supplementary material for: Fibroblast growth factor signalling influences homologous recombination-mediated DNA damage repair to promote drug resistance in ovarian cancer
Source: Br J Cancer. 2022 Jul 1;127(7):1340–51. doi: 10.1038/s41416-022-01899-z (PMC9519926; doi:10.1038/s41416-022-01899-z)
Supplement: Supplementary file 1 — Supplementary Information [file 41416_2022_1899_MOESM1_ESM.docx]

**Supplementary Information: Nicholson, H.A. *et al.* (2022).**

**Fibroblast growth factor signalling influences homologous recombination mediated DNA damage repair to promote drug resistance in ovarian cancer.**

**Supplementary Method 1 – Clonogenicity Assays.**

To assess clonogenicity in response to drug treatment, 1000 cells were seeded in 2mL RPMI per well of 6-well plates and incubated overnight. The following day, a dose range of cisplatin (0-25.33µM) or carboplatin (0-85.12µM) was added to each well, representing typical patient peak plasma concentrations (Konecny, 2000). Cells were incubated for 72h in drug then washed in 2mL PBS before incubation for a further 14 days in RPMI with 10% v/v FBS. Colonies formed were fixed by removing media and the addition of 1mL/well MeOH for 30 min. MeOH was removed and 1mL crystal violet stain (0.5% w/v crystal violet, 25% v/v MeOH, Sigma Aldrich, Dorset, UK) added per well for 15 min. Stain was then removed and plates gently washed with water to remove any excess stain before air drying. Images were taken of each plate using an iRADv5325 photocopier unit (Canon, Toyko, Japan) and colonies quantified using Fiji Image J (Schindelin, J. *et al.,* 2019). Clonogenic survival is represented as a percentage of vehicle treated control using GraphPad Prism 9 (GraphPad Software, CA, USA).

**Supplementary Method 2 – Flow Cytometry Analysis**.

1 x 10 ^6^ cells were plated in triplicate 10cm dishes and left to adhere overnight before harvest using 0.25% trypsin. Cell pellets were re-suspended in 1ml PBS + 1%FBS, transferred to FACS tubes (Scientific Laboratory Supplies, cat no: 352058) and pelleted following centrifugation at 1200rpm for 3 minutes. 1ml of ice cold 90% MeOH in dH_2_O was added to each cell pellet while vortexing, and cells fixed for at least 30 min at room temperature prior to storage at -20^o^C. Approximately 1 x 10^6^ cells per sample were then washed twice in PBS + 1% v/v FBS, pelleted by centrifugation at 1200 rpm for 3 minutes, re-suspended in 300μl staining buffer (50 μg/ml propidium iodide, 50 μg/ml ribonuclease A in PBS + 1% FBS) and incubated at room temperature protected from light for 20 min, prior to being analysed for DNA content using an LSR Fortessa (Becton Dickinson) and FACS DIVA acquisition software. Propidium iodide (PI) fluorescence was determined using 561nm excitation and collection of fluorescence at 582/15nm. Cell cycle distribution was determined using Flowjo software (Becton Dickinson). PI-Area and PI-Width measurements were used to exclude doublets and PI-A used to determine DNA content. Cell cycle distribution was calculated using the Watson Pragmatic model. All analysis was performed in triplicate and the experiment was repeated 3 times.

**Supplementary Figure 1**

**Genetic events in selected commonly mutated DNA repair genes in A2780, SK-OV-3 and CaOV3 ovarian cancer cell lines.**

A2780, SK-OV-3 and CaOV3 cell lines were assessed for mutations in commonly mutated DNA damage response pathway genes by searching for selected genes in the Broad Institute ‘depmap’ data explorer resource. The depmap contains a genome-scale catalogue of genetic vulnerabilities in many cancer cell lines, and can be found at depmap.org/portal.

**Supplementary Figure 2**


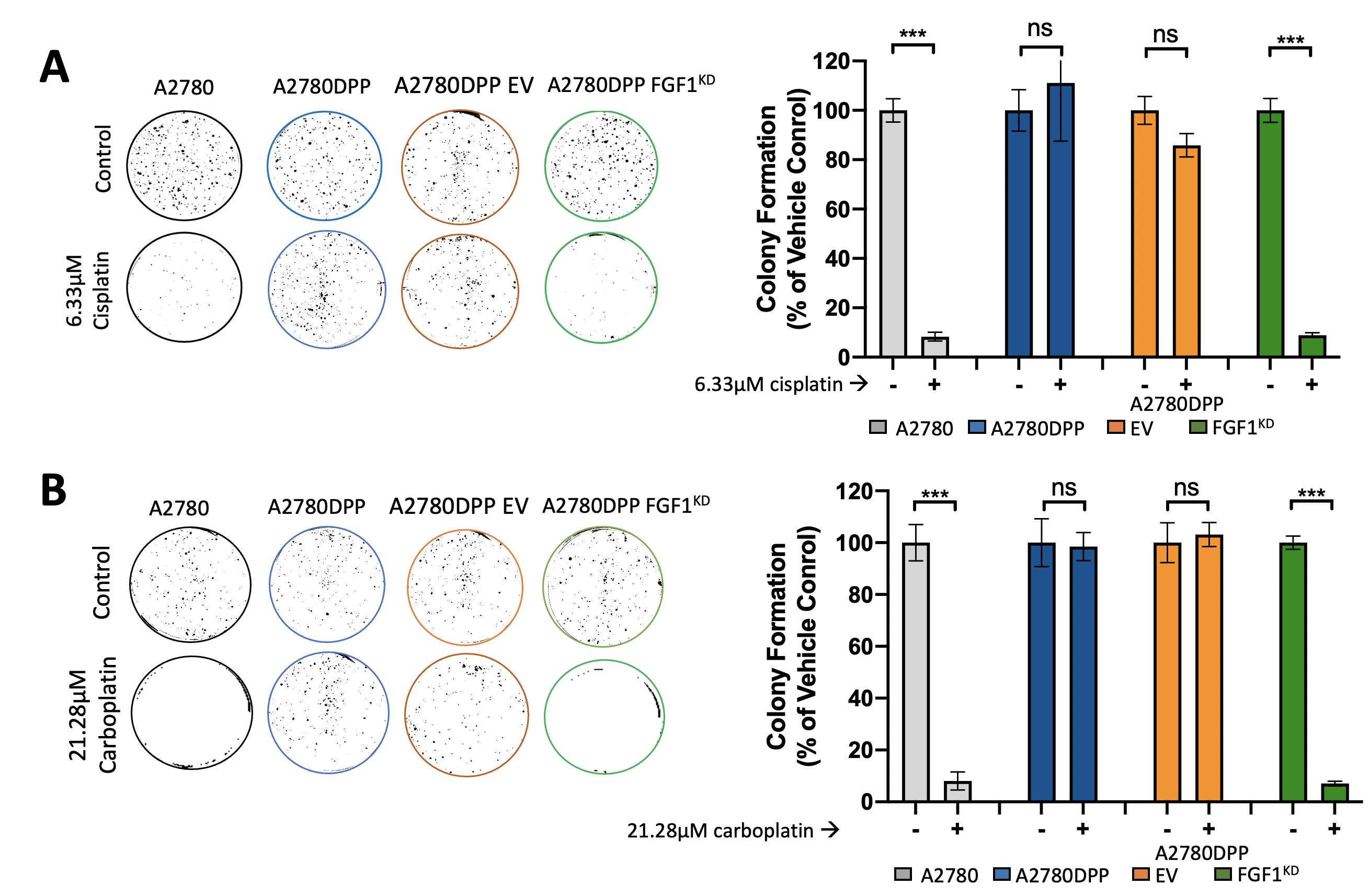


Knockdown of *FGF1* in A2780DPP cells reduced the ability of cells to form colonies in the presence of cisplatin or carboplatin.

Clonogenicity assays were used to investigate the colony forming ability of A2780, A2780DPP, A2780DPP EV and A2780DPP FGF1^KD^ cells in the presence or absence of cisplatin or carboplatin. Cells were grown in a dose range of cisplatin (0-25.33µM) or carboplatin (0-85.12µM) for 72 hours. Colonies were counted using Fiji Image J after 14 days, and colony formation calculated as a percentage of vehicle treated control cells. Representative colony formation and quantification of 50% peak plasma dose (and vehicle control) is illustrated for **A**) cisplatin and **B**) carboplatin. Error bars represent standard deviation. Data is representative of three independent experiments. Differences in mean clonogenic survival were compared by Students’ unpaired t tests. *** p<0.001. ns=not significant.

**Supplementary Figure 3**

**FGF1 induces resistance to cisplatin and carboplatin in chemonaïve ovarian cancer cells**

*FGF1* was overexpressed in **A**) SK-OV-3 cells and **B**) CaOV3 cells and green fluorescence imaging used to confirm FGF1 expression in transfected cells. **C**) Cells were cultured for 72hrs to increase cell numbers and FGF1 expression in transfected cells confirmed by Western blot analysis.n MTT assays were used to assess the influence of *FGF1* overexpression in SK-OV-3 cells on cell sensitivity to a dose-response of **C**) cisplatin (0=25.33µM) and **D**) carboplatin (0-85.12µM) and in CaOV3 cells to a dose-response of **D**) cisplatin (0-25.33µM) and **E**) carboplatin (0-85.12µM). Results are illustrative of three repeat experiments. Pairwise comparisons of mean IC_50_ values and relative gene expression were calculated by Students’ t tests. n=1. Scale bar = 100µm. *p<0.05, **p<0.01, ***p<0.001.

**Supplementary Figure 4**

**FGF1 induces RAD51 recombinase foci formation in response to cisplatin.**

Red fluorescence imaging (EVOS FLII microscope, ThermoFisher Scientific, Renfrewshire, UK) was used to compare RAD51 recombinase foci formation in response to acute cisplatin treatment over a 24h time-course in A2780, A2780DPP and A2780DPP FGF1^KD^ cells. Cells were pre-treated with 2mM thymidine for 24h to synchronise cells, before treatment with 3µM cisplatin over a 24h time-course, with RAD51 recombinase foci observed at the indicated timepoints. DAPI (blue) was used as a nuclear stain. Scalebar = 100µm. n=1.

**Supplementary Figure 5**

**Cisplatin resistance is associated with an FGF1-dependent G2 arrest**

Flow cytometry analysis was used to compare cell cycle parameters in untreated unsynchronised A2780, A2780DPP and A2780DPP FGF1^KD^ cells. A typical (A) scatter plot, gating strategy and quantitative analysis of cell cycle distributions is illustrated. Cell cycle profiles, analysed in triplicate in each cell line are illustrated in three independent experiments (B-D), with combined data and associated errors summarised in (F).

**Supplementary References**

(Konecny *et al.*, 2000)

(Schindelin *et al.*, 2012)

**1.** Konecny, G., Crohns, C., Pegram, M., Felber, M., Lude, S., Kurbacher, C., Cree, I. A., Hepp, H. & Untch, M. (2000). Correlation of drug response with the ATP tumorchemosensitivity assay in primary FIGO stage III ovarian cancer. *Gynecol Oncol,* **77:** 258-63.

**2.** Schindelin, J., Arganda-Carreras, I., RFrise, E., Kaynig, V. & al, e. (2012). Fiji: an open-source platform for biological-image analysis. *Nature Methods,* **9:** 676-782.
